# Effectiveness and safety of multiple injections of human placenta-derived MSCs for knee osteoarthritis: a nonrandomized phase I trial

**DOI:** 10.1186/s12891-025-08664-2

**Published:** 2025-04-26

**Authors:** Yevhen Holiuk, Roman Birsa, Tetiana Bukreieva, Petro Nemtinov, Vitalii Kyryk, Alina Ustymenko, Vadym Mazevych, Mykola Sokolov, Galyna Lobyntseva, Volodymyr Shablii

**Affiliations:** 1 State Institution “The Institute of Traumatology and Orthopedics by NAMS of Ukraine”, 27 Bulvarno-Kudriavska Street, Kyiv, 01601 Ukraine; 2 Department of Traumatology, Kyiv City Clinical Hospital, #6, 3 Guzara Ave, Kyiv, 03680 Ukraine; 3 Laboratory of Biosynthesis of Nucleic Acids, Institute of Molecular Biology and Genetics of National Academy of Science of Ukraine, 150 Zabolotnogo Str, Kyiv, 03143 Ukraine; 4 Placenta Stem Cell Laboratory, Institute of Cell Therapy, 9 Mokra str, Cryobank, Kyiv, 03035 Ukraine; 5 Institute of Cell Therapy, 9 Mokra str, Kyiv, 03035 Ukraine; 6 Cell and Tissue Technologies Department, M. D. Strazhesko National Scientific Center of Cardiology, Clinical and Regenerative Medicine of the National Academy of Medical Sciences of Ukraine, 5 Svyatoslav Khorobrygo str, Kyiv, 03151 Ukraine; 7 Laboratory of Pathological Physiology and Immunology, D. F. Chebotarev Institute of Gerontology of the National Academy of Medical Sciences of Ukraine, 67 Vyshgorodska Street, Kyiv, 04114 Ukraine; 8 Department of Protein Synthesis Enzymology, Institute of Molecular Biology and Genetics of National Academy of Science of Ukraine, 150 Zabolotnogo Str, 03143 Kyiv, Ukraine

**Keywords:** Knee osteoarthritis, Human placenta-derived mesenchymal stem cells, MSCs, Hyaluronic acid, Inflammation, WOMAC, VAS

## Abstract

**Objective:**

This study investigates the safety and efficacy of three intra-articular (IA) injections of cryopreserved human placenta-derived mesenchymal stem cells (hP-MSCs) for knee osteoarthritis (KOA) over a 1-year follow-up period.

**Methods:**

A total of 26 patients with stage II-III KOA were enrolled in this non-randomized, open-label study. Patients received either conventional therapy with hyaluronic acid (HA) alone (Control group, *n* = 11) or in combination with hP-MSCs (MSC group, *n* = 15) via three intra-articular injections with 4-week intervals. Clinical outcomes were assessed using the Western Ontario and McMaster Universities Osteoarthritis Index (WOMAC), Visual Analogue Scale (VAS), and magnetic resonance imaging (MRI) at 6 and 12 months following the first injection. Blood samples were analyzed for cytokine levels.

**Results:**

Three injections of hP-MSCs combined with HA were well-tolerated, with no severe adverse events observed. Significant improvements in WOMAC and VAS scores were noted in the MSC group compared to the Control group at both 6 and 12 months. MRI analysis revealed no significant differences in cartilage thickness or optical density index between the groups. Additionally, serum cytokine analysis showed a significant decrease in interleukin-2 (IL-2) levels in the MSC group, indicating an anti-inflammatory effect of hP-MSCs. However, no significant changes were observed in other cytokines.

**Conclusion:**

This study demonstrates that three intra-articular injections of cryopreserved hP-MSCs in combination with HA are safe and effective for treating KOA, providing sustained clinical improvement at the 1-year follow-up.

**Trial registration:**

NCT04453111, #7/09.26.2018. Registered 02 January 2020, https://www.clinicaltrials.gov/study/NCT04453111.

**Supplementary Information:**

The online version contains supplementary material available at 10.1186/s12891-025-08664-2.

## Introduction

Osteoarthritis (OA), a degenerative joint condition characterized by cartilage disintegration and underlying bone abnormalities, significantly impairs the quality of life for millions of people throughout the world. OA affects an estimated 528 million people globally, marking an increase of 113% since 1990. Approximately 73% of individuals with OA are over 55 years old, and 60% are female [1]. The knee, with a prevalence of 365 million, is the most commonly affected joint, followed by the hip and the hand, with a lifetime risk of knee OA (KOA) estimated at 45% [2, 3].

In addition, a high body mass index (BMI) contributes to 20.4% of osteoarthritis cases [4]. As the aging population grows and the rates of obesity and injury increase, these numbers are expected to rise, highlighting the need for a paradigm shift in the management of this condition. Osteoarthritis is influenced by various risk factors, with obesity playing a significant role in both weight-bearing and non-weight-bearing joints. Obesity contributes to OA through mechanical overload and systemic inflammatory mediators from adipose tissue, like adipokines and free fatty acids. Studies indicate a higher incidence of OA in obese individuals with cardiometabolic issues compared to “metabolically healthy” obese individuals, leading to the recognition of metabolic syndrome-associated OA [5]. Metabolic disorders, including diabetes, dyslipidemia, and hypertension further increase the risk of OA [6]. Some studies have found no strong link between OA and metabolic syndrome after adjusting for BMI, highlighting the complexity of OA beyond simple risk factor categorization [7–9].

Traditional knee OA treatments include pharmacological and surgical interventions, which often provide only symptomatic relief. Intra-articular treatments such as corticosteroids, hyaluronic acid (HA), and platelet-rich plasma (PRP) are widely used to manage knee OA. However, recent studies have raised concerns about the chondrotoxic effects of corticosteroids, which may potentially induce early-onset OA [10, 11]. While HA and PRP are commonly used, their effectiveness remains debated, and a consensus on their appropriate indications needs to be reached. Furthermore, these treatments typically offer short-term relief, lasting from several weeks to months.

Currently, arthroplasty is the only curative therapy for severe KOA, though it carries significant risks such as infection, residual pain, and stiffness. However, emerging strategies, particularly the use of mesenchymal stem cells (MSCs), offer a potential alternative to OA therapy. MSCs as multipotent stromal cells with the ability to differentiate into osteoblasts, adipocytes, and chondrocytes, exhibit high plasticity, self-renewal capabilities, and immune-suppressive and anti-inflammatory properties.

Clinical studies using MSCs emphasize the importance of distinct origins, with bone marrow- and adipose-derived MSCs being intensively researched for knee OA therapy [12–14]. In contrast, human placenta-derived MSCs (hP-MSCs), a relatively new source, offer benefits such as increased proliferative potential without the ethical concerns associated with other sources.

Despite this, only limited number of clinical trial results have been published regarding the use of hP-MSCs in KOA treatment. Broader criteria for prescribing intra-articular cell therapies, including methods of MSCs isolation, number of injections and cell dose, administration strategy, and outcome registration remain unclear [15–17]. The majority of published research has focused on a single injection of MSCs, with only a few studies examining the efficacy of two injections of allogeneic human umbilical cord-derived MSCs (hUC-MSCs) in combination with HA [18], and one study exploring four injections [19].

Our study focused on the effect of three intra-articular injections of cryopreserved human placenta-derived MSCs combined with hyaluronic acid on KOA.

## Methods

### Participants and study design

A non-randomized, open-label study was conducted at the Kyiv City Clinical Hospital #6 and State Institution (SI) “The Institute of Traumatology and Orthopedics by NAMS of Ukraine” from February 2020 to June 2022. The study protocol was designed following the Declaration of Helsinki and approved by both the Ethics Committee of Kyiv City Clinical Hospital #6 (protocol #2256, July 11, 2018) and the SI “The Institute of Traumatology and Orthopedics by NAMS of Ukraine” (protocol #4, July 11, 2018). The study was approved by the Coordination Center for Organ, Tissue, and Cell Transplantation of the Ministry of Health of Ukraine (protocol #7, dated September 26, 2018), and it was registered on ClinicalTrials.gov (NCT04453111).

A total of 32 patients (11 men and 21 women, aged 23 to 78 years old) were initially enrolled in the study. At the initial stage, 6 patients (2 men and 4 women) were excluded based on the exclusion criteria or because they did not meet the inclusion criteria. The inclusion criteria were as follows: a verified clinical diagnosis of stage II–III knee osteoarthritis according to Kellgren & Lawrence classification based on X-ray data, chronic joint pain associated with mechanical factors characterized by persistence and fluctuations in intensity lasting three months or longer, and age 18–75 years. The exclusion criteria included the following: local or systemic infection, use of oral or intra-articular corticosteroids or anticoagulants, previous malignancy or organ transplantation, history of stroke or myocardial infarction, kidney or liver failure, or age < 18 or > 75 years. All participants enrolled in the study signed informed consent statements.

The 26 selected patients (9 men and 17 women, aged 23 to 74 years old) were divided into two groups based on the treatment method. Eleven patients who received a three-dose treatment with HA (Hyalgan^®^, 20 mg/2 ml; Fidia Farmaceutici S.P.A.) at 4-week intervals were assigned in the Control group. In the MSC group, fifteen patients, in addition to receiving conventional therapy with HA, were treated with three consecutive doses of hP-MSCs. There were no significant differences between the two groups in terms of age (*P* = 0.146), sex (*P* = 0.61), BMI (*P* = 0.285), or knee OA grade (*P* = 0.624). Table 1 presents the results of the investigations of the variables and demographic characteristics.

**Table 1:** Clinical characteristics of patients with KOA included in this study

Parameter	Control group (*n* = 11)	MSC group (*n* = 15)	*p* value
Age, years, mean (range)	61.5 (32–69)	53.9 (24–72)	0.146
Gender, n (%)			0.61
Male Female	4/11 (36.36%) 7/11 (63.64%)	5/15 (33.33%) 10/15 (66.67%)	
BMI (kg/m^2^), mean (± SD)			0.285
< 30 > 30	28.3 (± 1.5) 6 5	30.4 (± 1.2) 7 8	
Kellgren grade, n (%)			0.624
II III	3/11 (27.27%) 8/11 (72.78%)	5/15 (33.33%) 10/15 (66.67%)	
WOMAC, mean	35.7	31.0	0.502
VAS 0–100, mm, mean	4.77	4.74	0.96

### hP-MSCs preparation

**Fig. 1:**
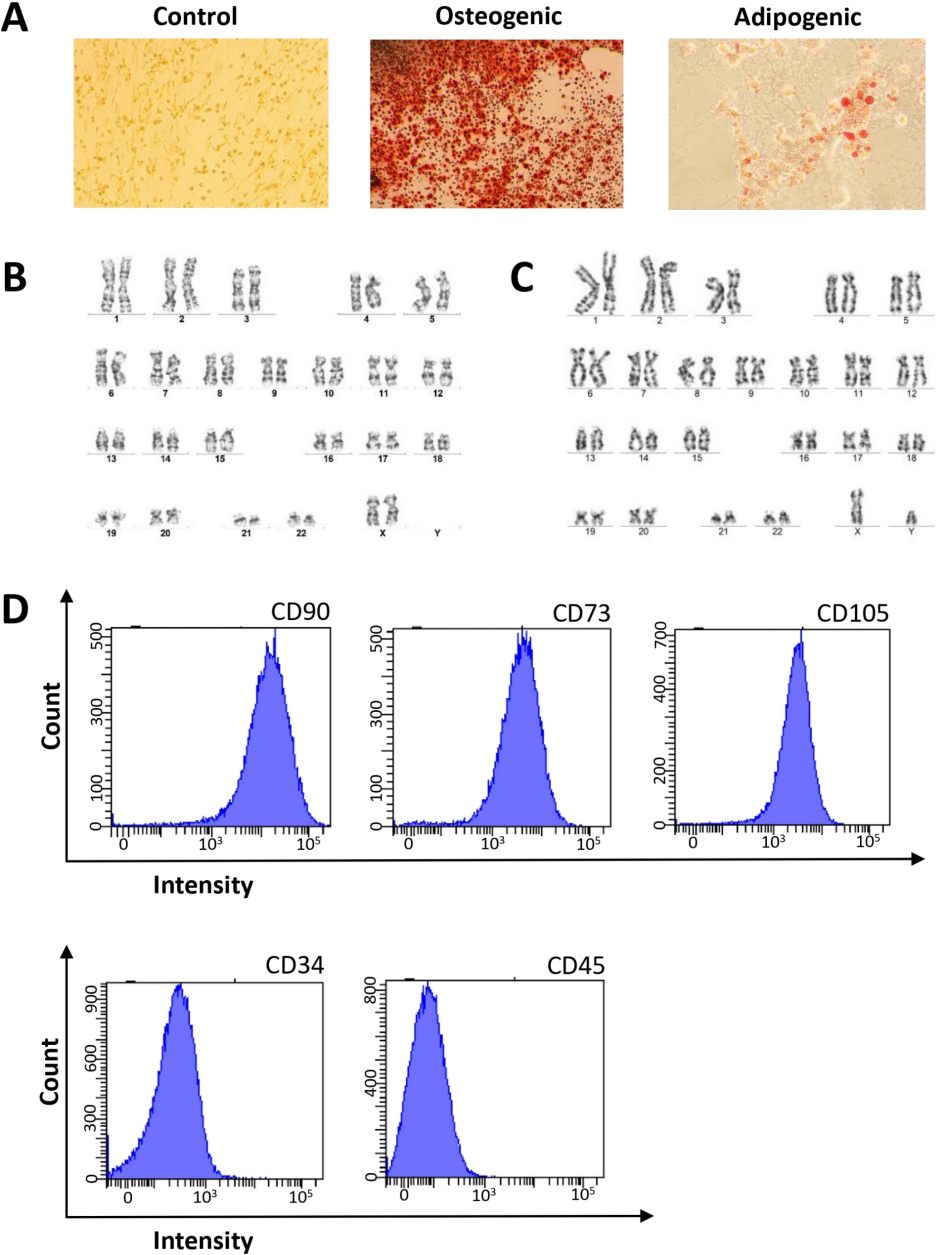
Characteristics of hP-MSCs. **A**, Abundance of lipid vacuoles and single calcifying nodules in the extracellular matrix were detected in hP-MSC cultures at P3 after adipogenic and osteogenic induction; Oil Red O and Alizarin Red S staining respectively, 50×. **B**, **C** representative example of a normal female (**B**) and male (**C**) karyotype of a hP-MSCs line at P3. **D**, hP-MSCs were positive for CD90, CD73, and CD105 (≥ 95%) and negative for CD34 and CD45 (≤ 2%) according to flow cytometry data at P3

The placentas obtained after Caesarean section were collected from donors aged 23 to 36 years at 39–41 weeks of gestation from Kyiv City Maternity Hospital #3. All donors (*n* = 9) provided written informed consent for the inclusion of their placentas in the approved clinical study. One week prior to placenta collection, apparently healthy donors underwent screening for infectious diseases using serological assays (anti-HIV1/2, anti-HCV, anti-HBV, anti-Treponema pallidum, and anti-CMV IgG and IgM) and qRT–PCR (to detect nucleic acids of *HIV1/2*, *HCV*, and *HBV*) in a licensed external laboratory. Validated PCR kits were used to test placental tissues for *HSV-1/2*, *HHV-6*, *Ureaplasma* spp., and *Mycoplasma genitalium* in the PCR laboratory in the Institute of Cell Therapy. Under aseptic conditions in a biosafety cabinet, the amnion was excised, and a 25-gram fragment of the chorionic plate and chorionic villus (3–7 mm thick) was dissected using sterile scissors. The placental tissues were then minced into small pieces (1–3 mm) and vigorously washed on a shaker in Hanks’ balanced salt solution (HBSS) (Sigma, Irvine, UK) supplemented with 100 U/mL penicillin (Arterium, Kyiv, Ukraine) and 50 mg/mL streptomycin (Arterium, Kyiv, Ukraine) until the washing solution became colorless. Subsequently, the tissue fragments underwent digestion with 0.1% collagenase type I (Serva, Germany) and 0.6 U/ml dispase I (Gibco, USA) in 5 ml of DMEM (HyClone, USA) supplemented with 5 mM HEPES (MP Biomedicals, USA). Semidigested tissue pieces were seeded into 175-cm^2^ tissue culture flasks (Sarstedt) in alpha-MEM (HyClone, USA) supplemented with 15% FBS (HyClone, USA), 1× RPMI amino acid solution (Sigma, USA), and 1× streptomycin/penicillin (Sigma, USA) to complete the culture medium. The explants were incubated at + 37 °C with 5% CO2 for 14 days, after which the medium was changed twice a week. Upon reaching 80–90% confluence, the outgrown cells were detached using 0.05% trypsin and 0.02% EDTA (Sigma, Irvine, UK), washed, counted, and passaged at a seeding density of 4–5 × 10^3^ cells/cm^2^ in culture-treated flasks, referred to as passage 1 (P1). hP-MSCs at passage 3 (P3) were harvested and cryopreserved using a rate-controlled freezer supplemented with 5% dimethyl sulfoxide (Sigma Aldrich, Saint Louis, MO, USA) in HBSS (Sigma, Irvine, UK) at a final concentration of 5 × 10^6^ cells/ml. Quality control measures, including viability assessment using the Trypan Blue exclusion method, expression of cell surface markers via flow cytometry, directed differentiation into osteogenic and adipogenic lineages, cytogenetic analysis using the GTG-banding method, and microbiological tests (Bact/Alert 3D, Biomerieux, Durham, NC, USA), were performed on aliquots from all samples at passage 3 as described previously [20]. *Mycoplasma* spp. detection was carried out using the MycoAlert™ PLUS Mycoplasma Detection Kit (Lonza, Rockland, Miami, FL, USA) following the manufacturer’s instructions. These stringent quality control procedures were performed before each batch of cells was released for further use. Representative images of proliferating hP-MSCs, surface immunophenotype, directed differentiation assay, and karyotype analysis are presented in Fig. 1.

### hP-MSCs transplantation

For injection, cryopreserved hP-MSCs at P3 were thawed in a water bath heated to + 37 °C until the liquid phase formed. The cells were centrifuged at 300 ×*g* for 5 min at room temperature, and the resulting cell pellets were resuspended in a final volume of 2 mL of a 0,9% saline vehicle solution (Darnytsya, Kyiv, Ukraine) supplemented with 5% human serum albumin (HSA) (Biopharma, Kyiv, Ukraine). Cell viability was assessed using the Trypan Blue exclusion test prior to injection, with the average viability reaching 85.4 ± 4.3%. The cells in 2 ml of vehicle solution were mixed in 5 mL syringe with 2 mL of Hyalgan^®^ containing 20 mg of HA and injected using an anterolateral approach at to the medial joint cavity line with the knee flexed at 90° The final cell count was 20 × 10^6^ per injection. The total treatment dose over the course of three injections was 60 million cells.

### Follow-up

**Fig. 2:**
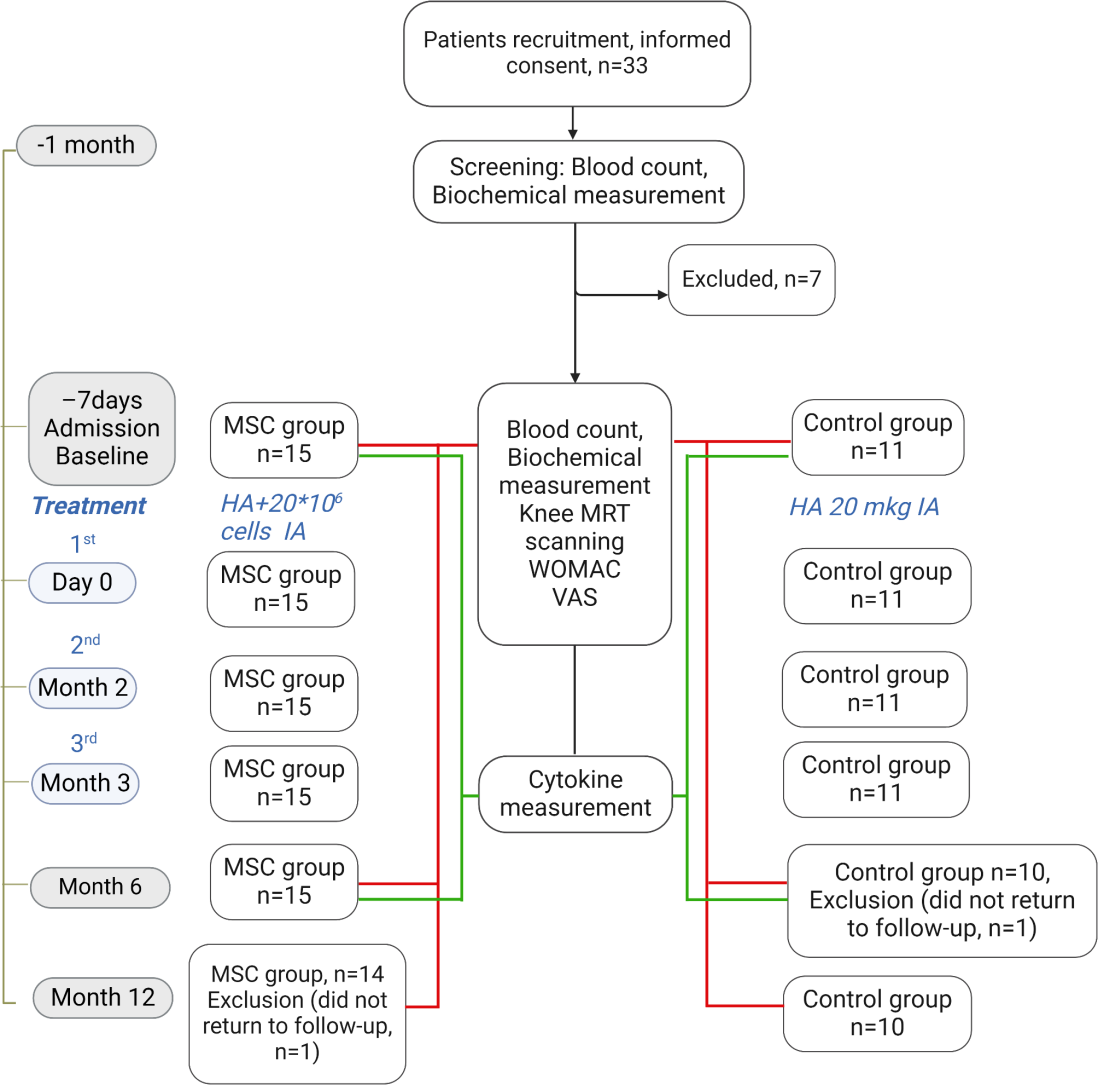
Flow chart for patient enrollment, intervention and follow-up. Abbreviations: HA, hyaluronic acid; MSC, mesenchymal stromal cell; MRI, magnetic resonance imaging; IA, intra-articular; WOMAC, Western Ontario and McMaster Universities Arthritis Index; VAS, visual analog scale

Clinical outcomes and trial assessments were evaluated at 6 and 12 months following the first injection by the same surgeon involved in the treatment procedures. The 1-year follow-up period was completed by 24 patients, and one patient from each group not returning (Fig. 2). To assess knee OA, we used the Western Ontario and McMaster Universities Osteoarthritis Index (WOMAC) and Visual Analogue Scale (VAS) to evaluate symptoms such as pain, stiffness, and functional disability. In addition to the orthopedic clinical examination, we dynamically assessed cartilage parameters using knee joint magnetic resonance imaging (MRI) with contrast. We also measured cytokine levels, including Interleukin 2 (IL-2), Interleukin 10 (IL-10), Interferon gamma-induced Protein 10 (IP-10), Macrophage Inflammatory Protein-1 Alpha (MIP-1α), Tumor Necrosis Factor Alpha and Monocyte Chemoattractant Protein 1 (MCP-1) in peripheral blood using enzyme-linked immunosorbent assay (ELISA). During the treatment period, we also monitored and assessed the frequency and nature of each adverse events (AEs) to determine whether they were related to the administration of hP-MSCs.

### Indices determination

We used the WOMAC questionnaire as a standardized tool to assess symptoms of knee and hip osteoarthritis. The questionnaire includes items related to pain, stiffness, and physical, emotional, and social function, with each question scored on a scale from 0 to 4 points [21].

VAS was used as a validated, subjective measure for acute and chronic pain. Patients marked their pain level on a 10-cm line, which represents a continuum from “no pain” to “worst pain” [22]. WOMAC and VAS were assessed at baseline (before treatment), and again at 6 and 12 months after the administration of HA and hP-MSCs.

Body mass index was calculated by dividing body weight (kg) by height squared (m^2^).

### MRI examination

Knee MRI assessments were performed at baseline, 6 months, and 12 months using a Philips Achieva 1.5 T (Philips Healthcare, Netherlands) MRI machine. The study was utilized intra-articular contrast enhancement with 0.5 ml of gadoteric acid (Dotavist, Farmak, Ukraine) and 10 ml of 0.9% saline. The following indices were assessed: cartilage thickness and optical density of cartilage tissue. Cartilage thickness was measured at 15 points: Patella– P1, P2, P3; Tibia Lateral– TL1, TL2, TL3; Tibia Medial– TM1, TM2, TM3; Femur Lateral– FL1, FL2, FL3; and Femur Medial– FM1, FM2, FM3. Additionally, the difference (delta of increase) between the 6 and 12-months measurements and the baseline values was calculated for each patient to adjust individual characteristics. The optical density of the cartilage tissue was measured in 3D WATS post-contrast in the P, TL, TM, FL, and FM areas, and the soft tissue proportionally related to cartilage hydrophilicity. To standardize measurements across different device settings, the optical density index was calculated in each area (P/S, etc.) relative to the optical density of soft tissues (S).

### Blood collection

Peripheral venous blood samples (12–20 mL) were collected from 26 patients with KOA into BD Vacutainer^®^ blood collection tubes containing K_3_EDTA for plasma or clot activator, silicone coated tubes (Becton Dickinson, USA) for serum on the day of admission (baseline) and six months after admission. Briefly, 5 mL was used for routine blood assays completed using a Swelab Alfa Basic hematology analyzer (Boule Medical AB, Spånga, Sweden) and for the determine of erythrocyte sedimentation rate by Panchenko’s method in the Clinical Diagnostic Laboratory of the Institute of Cell Therapy. The plasma and serum were separated from the remaining blood samples, snap-frozen, and stored at − 80 °C for subsequent cytokine detection. Uric acid and ALT were determined by an enzymatic colorimetric technique (PAP method) using validated kits according to the manufacturer’s instructions (HUMAN Gesellschaft für Biochemica und Diagnostica mbH, Germany). Detection of antistreptolysin O and rheumatoid factor was performed at a licensed external laboratory.

### Cytokine measurement

To detect IL-2, IP-10, MIP-1α, IL-10, TNF-α and MCP-1, an enzyme-linked immunosorbent assay (ELISA) was performed using an Invitrogen kit according to the manufacturer’s instructions. The following ELISA kits and standard curves were used to measure each parameter: human IL-2 (BMS221INST), IP-10 (BMS284INST), MIP-1α (KAC2201), IL-10 (BMS215INST), TNF-α (BMS223HS) and MCP-1 (BMS281INST) from Instant ELISA (Invitrogen, Thermo Fisher Scientific, Vienna, Austria). The sensitivity levels for each assay were 2.3 pg/mL for IL-2, 1 pg/mL for IP-10, 2 pg/mL for MIP-1α, 0.66 pg/mL for IL-10, 0.13 pg/mL for TNF-α and 2.31 pg/mL for MCP-1. All absorbance measurements were carried out using a HumaReader HS (Human GmBH, Wiesbaden, Germany) microplate reader. All assays were performed in duplicate.

### Statistical analysis

The results are presented as median values with interquartile range. Statistically significant differences between the groups were determined using the independent samples *t*-test. The paired *t*-test was applied to compare time-dependent events within groups. Statistical significance was set at a two-tailed *p*-value ≤ 0.05. Statistical analyses were performed using STATISTICA 8.0 software (StatSoft Inc. 2007, USA). GraphPad Prism software (version 7.0a, Inc. San Diego, CA, USA) was used for data visualization.

## Results

**Fig. 3:**
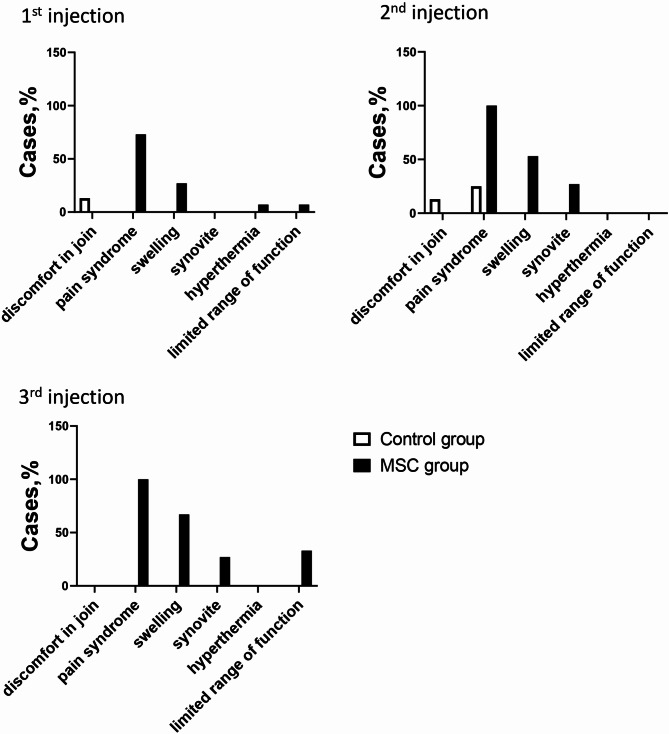
Adverse events occurred in the groups after each injection

In the MSC group, joint pain was reported by 73% and swelling by 26.7% of patients after the first injection. In contrast, according to self-report data, only 12.5% of patients in the Control group reported discomfort. It is important to note that after the second and third injections of hP-MSCs, there was a significant increase in the percentage of patients with pain syndrome, swelling, and synovitis, as well as those with limited mobility (Fig. 3), compared to the Control group. All of these AEs were resolved safely within 3–7 days. No serious adverse effects were observed at 2 weeks post-injection.

**Fig. 4:**
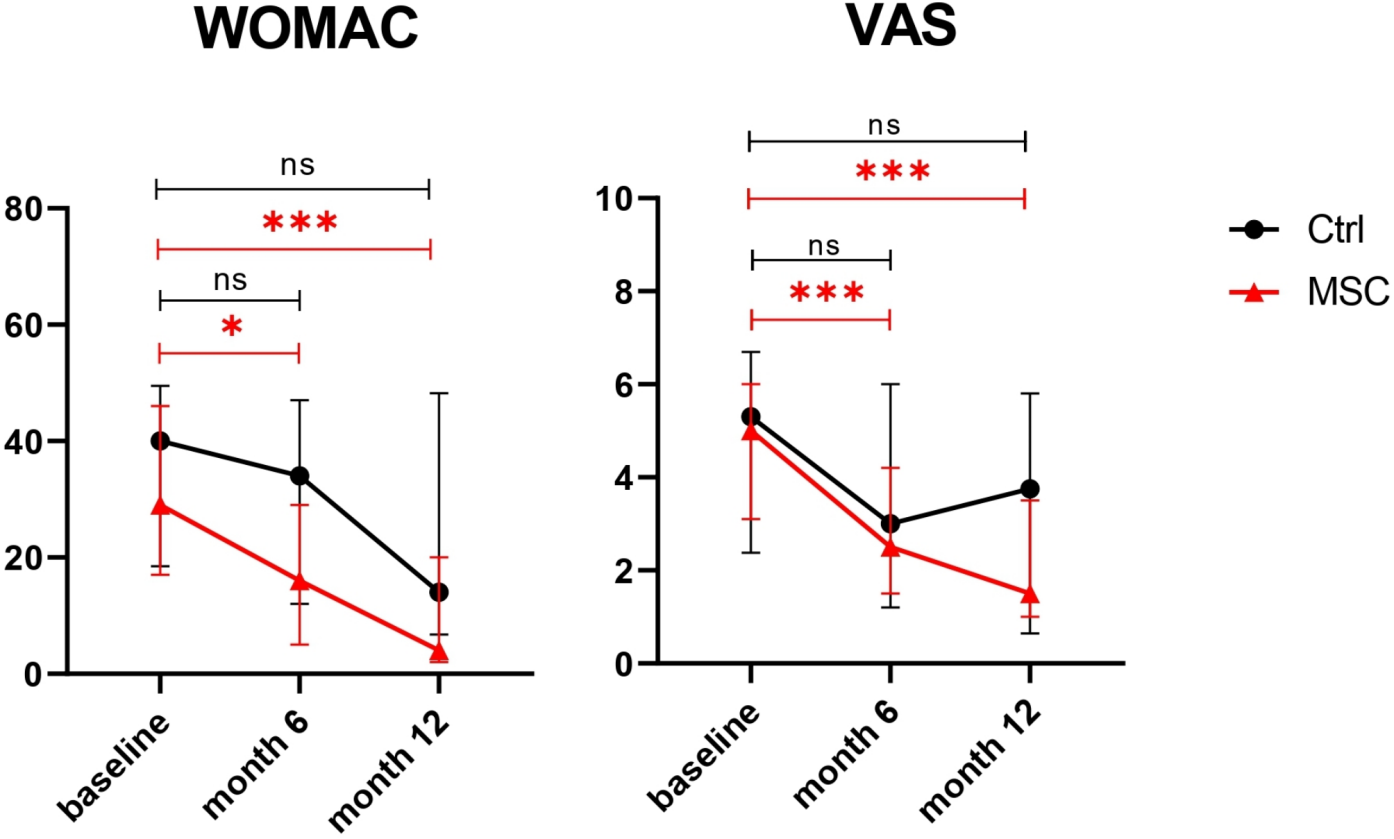
Changes in the scores of each group. The black line with dots represents the Control group; the red line with triangles represents the MSC group. *, *p* ≤ 0.05; ***, *p* ≤ 0.001; ns, not significant. Abbreviations: Ctrl, control group; MSC, mesenchymal stromal cells group; WOMAC, Western Ontario and McMaster Universities Arthritis Index; VAS, visual analog scale

In the MSC group, WOMAC and VAS scores were significantly lower at 6 months and one year after therapy compared to baseline. In contrast, these scores in the Control group showed no significant change over the 1-year follow-up. There was no significant difference in the WOMAC scores between the MSC and the Control groups at the 6-month and 1-year follow-up points (Fig. 4).

MRI analysis of knee joint cartilage thickness at various measurement points did not show significant differences between the groups one year after therapy (Supplementary Figures S1 and S2).

**Fig. 5:**
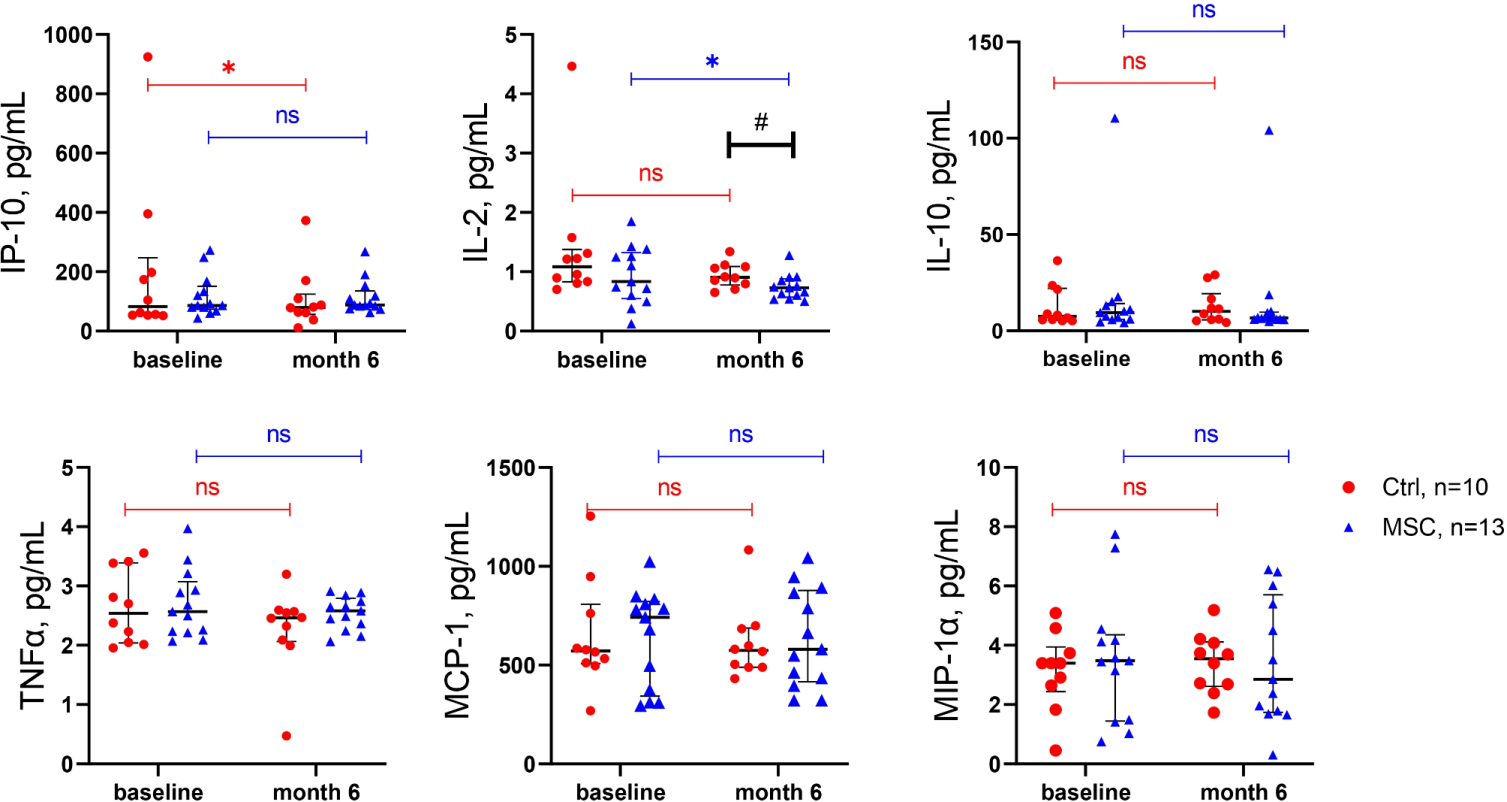
Serum cytokine levels in patients with KOA. *, *p* ≤ 0.05; #, *p* ≤ 0.05; ns, not significant. The red dots represent the Control group; the blue triangles represent the MSC group. IP-10, Interferon gamma-induced protein 10; IL-2, interleukin 2; IL-10, interleukin 10; TNF-α, tumor necrosis factor alpha; MCP-1, monocyte chemoattractant protein-1; MIP-1α, macrophage inflammatory protein-1 alpha

Blood laboratory tests were performed before and six months after treatment. As a result, patients from the Control group had a significant increase in the banded neutrophil count and percentage six months after HA treatment, while no such increase was observed in the MSC group. All other laboratory parameters remained unchanged (Supplementary Figure S3). The serum levels of cytokines TNF-α, IP-10, MIP-1α, MCP-1, and IL-10 did not differ between the groups or over the 6-month follow-up period. Notably, the IL-2 level in the MSC group significantly decreased after six months, and this decrease was significantly greater than that observed in the Control group (Fig. 5).

## Discussion

This trial compared the safety and efficacy of three injections of hP-MSCs combined with HA versus intra-articular HA alone in patients with symptomatic knee OA. To the best of our knowledge, this is the first study to evaluate the effects of three consecutive injections of hP-MSCs on knee OA. However, previous studies have reported the impact of two injections of hUC-MSCs or adipose-derived MSCs [18, 23, 24]. In the study by Ao et al., the efficacy of repeated 4-time hUC-MSC injections was evaluated, but the interval between injections was only 1 week, with a total dose of 60 million cells [19]. No safety signals were detected in the experimental group compared to the HA Control group.

The release criteria for the clinical application of hP-MSCs included the absence of contamination with bacteria, mycoplasma, and fungi; a normal male or female karyotype; and purity patterns characterized as positive (≥ 95%) for CD73, CD90, and CD105 and negative (≤ 2%) for CD45 and CD34 expression of cell surface markers according to the minimal criteria for defining multipotent mesenchymal stromal cells issued by ISCT [25]. Over the past decade, there has been a growing diversification of MSC products, with a strong trend for perinatal derivatives to become the most popular source in the past 2 years [26]. The popularity of perinatal tissue as a source of MSCs is due to the absence of ethical restriction, lack of AEs related to surgical intervention for collection, high availability (as biological waste), and fetal origin, which helps preserve their high proliferative potential [27]. Additionally, the placenta is an immunosuppressive organ, and its stromal cells possess natural immunomodulatory properties [27], making them highly relevant for knee OA treatment. Both the umbilical cord and placental stromal component share the same ontological origin, derived from extraembryonic mesoderm [28]. Compared to the umbilical cord, the placenta offers the advantage of its larger size, which allows for the isolation of a greater number of MSCs. However, disadvantages include the potential risk of maternal cell contamination, necessitating additional verification during manufacturing. In recent years, the most widely used perinatal tissue cells for treating OA have been hUC-MSCs [29], while only one study was published dedicated to the application of placental MSCs [18]. A few reviews have reported results from eight to twelve clinical trials involving the treatment of OA with hUC-MSCs alone or in combination with HA [30, 31]. It should be noted that some clinical trials have used hUC-MSCs in combination with surgical procedures to treat knee OA [30]. On the other hand, most of the aforementioned clinical trials have been at phases 1 and 2, and only one study achieved phase 2/3 [32]. A review of the literature and meta-analysis of clinical trial results on the use of hUC-MSCs have shown that patients treated with hUC-MSCs for knee OA experience improved clinical outcomes [31, 33]. There are also studies on the effectiveness of using hUC-MSCs in combination with hyaluronate hydrogel, but they significantly differ in the methodology of cell transplantation directly into surgically created microfractures in the knee cartilage or in combination with high tibial osteotomy [34–37]. However, further high-quality randomized trials are necessary to more accurately assess the efficacy of hUC-MSCs for the treatment of knee OA.

The repeated knee injections of hP-MSCs in our study led to an increase in the incidence of AEs, specifically pain syndrome in the knee, swelling, and mobility limitations. These AEs were resolved within one week, and no severe adverse events (SAEs) were observed. Soltani et al. reported that intra-articular administration of placenta-derived MSCs resulted in mild effusion and increased local pain in several patients, which resolved safely within 48–72 h [34]. Notably, after two injections of hUC-MSCs, mild to moderate symptomatic knee effusion and pain were not reported in the study Matas J. et al. [18]. However, in the study by Samara O. et al., during ultrasound-guided intra-articular injection of hUC-MSCs, most AEs included mild pain in seven patients and moderate pain in five patients out of 16 [17]. This difference may be due to the longer interval (6 months) between repeated injections in the mentioned studies as well as the use of native cells that were administered after cryopreservation and grown for two passages. The second injection of MSC therapy at six months in the two-injection group was associated with a modest increase in reported moderate AEs compared to the initial injection [23]. Similar to our findings, the percentage of AEs (transient pain and swelling) was recorded in another clinical study using two injections of autologous adipose-derived MSCs with an interval of 2 weeks [24]. Despite these short-term AEs, which are common for MSC therapy, most studies and meta-analyses report a positive effect of MSCs on the progression of knee OA [15].

We observed improvements in the WOMAC and VAS scores at 6 and 12 months; however, these scores did not significantly differ from those of the HA group. The cumulative dose of hP-MSCs we selected represents an average dose based on previously published clinical studies that demonstrated effectiveness in the treatment of KOA [38–40].

Currently, one article has been published on the single administration of human placental MSCs in stage 2–4 KOA. This study reported clinical improvement was found two months after the start of treatment; however, no reliable effect was achieved after six months [41]. It is worth noting that Soltani et al. [41] used native hP-MSCs injected at a high passage number (p12), which is atypical in other studies and poses challenges to standardization and scalability under manufacturing conditions. It is important to note that while there is a substantial amount of diverse data on the effectiveness of MSCs in treating KOA [38], limited information exists on the effects of repeated administration of allogeneic MSCs. To date, a few clinical studies on the two administrations of MSCs have demonstrated positive results in terms of quality of life and reduction of pain syndrome (VAS, WOMAC) within a 1-year follow-up [23, 24, 42]. We were able to find only one study involving four intra-articular injections of hUC-MSCs administered at 1-week intervals, which demonstrated safety in treating OA and did not induce SAEs; however, the follow-up period was limited to 3 months [19]. Additionally, the simultaneous administration of MSCs and HA has been previously studied, confirming the safety and clinical as well as functional improvement of these treatments in patients with knee OA [43].

Most researchers agree that biomechanical, inflammatory, and metabolic factors play a crucial role in the onset and progression of OA. The OA metabolic phenotype, driven by widespread inflammation of adipose tissue, may be less responsive to local regenerative therapies [8]. A review of the literature and the results of our patients’ examinations confirm the presence of different osteoarthritis phenotypes, which have clinical significance and differ in their pathophysiology and disease progression [7, 44]. This lays the groundwork for patient stratification, individualized therapy selection, and the development of new personalized treatment approaches, including the use of cell therapy.

In our study, MRI analysis did not reveal any changes in the knee joint cartilage thickness at different measurement points one year after therapy in both the MSC and Control groups. The lack of influence on cartilage thickness of hUC-MSCs treatment was previously published in another clinical trial [32, 45]. On the other hand, several trials showed the MRI improvement in cartilage 1 year after hUC-MSCs therapy [31, 46]. It should be noted that the difference in MRI outcome following MSC therapy could depend on factors such as the cell dosage, frequency of administration and variance of baseline characteristic of knee OA in relatively small groups of patients. Additionally, it is possible that a 1-year follow-up period was too short for this secondary measure due to the slow progressive process of OA.

While OA is often considered a localized joint disease, emerging evidence suggests it also has systemic inflammatory components. Serum levels of IL-2, IP-10, MIP-1α, IL-10, and MCP-1 can reflect inflammation, providing insights into disease progression or remission and supporting personalized treatment plans. In our study, treatment of patients with KOA using hP-MSCs led to a significant decrease in serum IL-2 concentration compared to the Control group, indicating the anti-inflammatory effects of MSCs. IL-2 is known to be one of the main inflammatory cytokines and plays a significant role in KOA progression [47]. On the other hand, we did not observe an effect of hP-MSCs on the levels of IL-10, TNF-α, or the chemokines MCP-1, MIP-1α, and IP-10 in patient blood, despite the fact that the levels of cytokines and chemokines significantly increase during the inflammatory process that accompanies the development of KOA [48]. In the study Li J. et al., significant reductions in the serum TNF-α and IL-6 levels were detected in the autologous bone marrow-derived MSC-treated group at 6 and 12 months, compared to baseline and to the HA control group demonstrating the systemic anti-inflammatory effects of MSC injections [49]. Bastos et al. showed that there were no significant differences in the levels of the inflammatory cytokines TNF-α, IL-10, and IL-2 in the synovial fluid of patients with knee OA (K&L grade 1–4) treated with autologous MSCs, autologous MSCs plus autologous PRP, or corticosteroids at 6 and 12 months after treatment [50]. IL-10 induces the expression of gene network involved in chondroprotective, antiapoptotic, and anti-inflammatory effects by stimulating the synthesis of type II collagen and aggrecan, as well as the inhibiting of MMP synthesis [51, 52]. The concentration of TNF-α was increased in the synovial fluid, cartilage, and synovial membrane of OA joints. TNF-α promotes the production of pro-inflammatory molecules (iNOS, COX-2, IL-6, IL-8, MCP1, RANTES) in chondrocytes, matrix degrading proteases and suppresses the synthesis of type II collagen and aggrecan [53]. In addition, neutrophils produce IP-10 in inflamed synovial fluids, contributing to the localization and activation of NK cells and macrophages in the joints, which aids disease establishment [54].

Elevated levels of MCP-1 have been found in the synovial fluid of patients with both knee injuries and knee OA [55, 56]. MCP-1 increases MMP-3 expression, leading to proteoglycan loss and the degradation of cartilaginous tissue [57]. Plasma levels of MIP-1α in patients with knee OA increased with the radiographic severity of the disease [58].

Therefore, our study showed that three injections of allogeneic cryopreserved hP-MSCs in combination with hyaluronic acid were safe and resulted in substantiated clinical improvements in patients with KOA stages II-III. However, a larger clinical trial, including a comparator arm, would help define the most appropriate cell dose and frequency of hP-MSCs treatment administration.

## Conclusion

Multiple intra-articular injections of allogeneic human placenta-derived MSCs combined with hyaluronic acid are safe for treating knee osteoarthritis and appear to be effective at the 1-year follow-up. An advanced, dose-dependent, placebo-controlled, double-blinded clinical trial is needed to determine the efficacy of administering human placenta-derived MSCs to patients with knee osteoarthritis.

## Electronic supplementary material

Below is the link to the electronic supplementary material.

Supplementary Material 1

Supplementary Material 2

Supplementary Material 3

## Data Availability

The datasets supporting the conclusions of this article are included within the article and supplementary materials.

## Abbreviations

IA: Intra-articular
hP-MSCs: Human placenta-derived mesenchymal stem cells
hUC-MSCs: Human umbilical cord-derived mesenchymal stem cells
KOA: Knee osteoarthritis
WOMAC: Western ontario and mcmaster universities osteoarthritis index
VAS: Visual analog scale
AE: Adverse event
BMI: Body mass index
K&L: Kellgren & lawrence
NAMS: National academy of medical sciences
ELISA: Enzyme-linked immunosorbent assay
IP-10: Interferon gamma-induced protein 10
IL-2: Interleukin 2
IL-10: Interleukin 10
TNF-α: Tumor necrosis factor alpha
MCP-1: Monocyte chemoattractant protein-1
MIP-1α: Macrophage inflammatory protein-1 alpha
ASO: Antistreptolysin O
ALT: Alanine transaminase
RF: Rheumatoid factor
ESR: Erythrocyte sedimentation rate
