# Supplementary material for: Effectiveness and safety of multiple injections of human placenta-derived MSCs for knee osteoarthritis: a nonrandomized phase I trial
Source: BMC Musculoskelet Disord. 2025 Apr 26;26:418. doi: 10.1186/s12891-025-08664-2 (PMC12032682; doi:10.1186/s12891-025-08664-2)
Supplement: Supplementary file 1 — Supplementary Material 1 [file 12891_2025_8664_MOESM1_ESM.docx]

# **Supplementary materials**


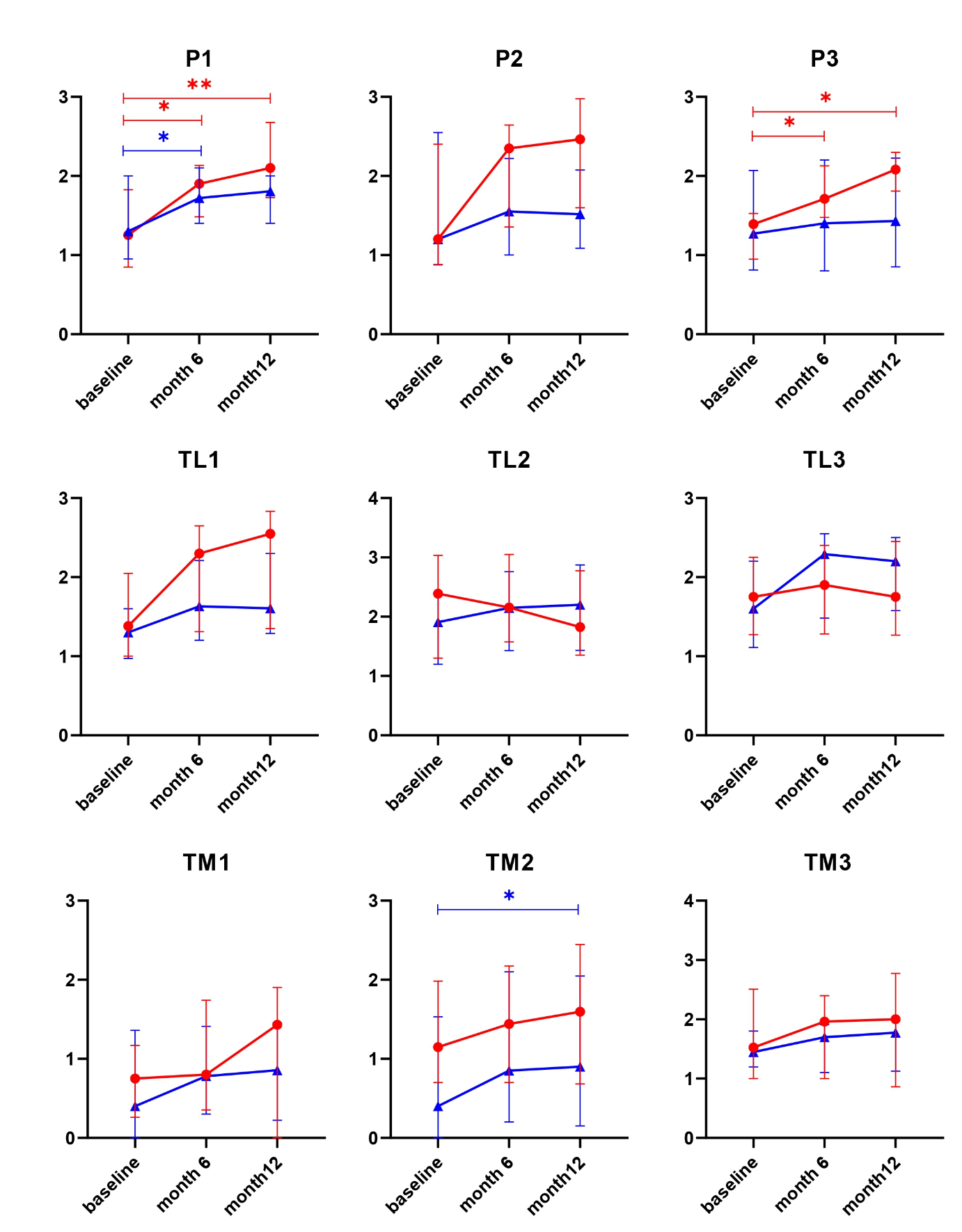


Figure S1. MRI analysis of cartilage thickness in patients with KOA at different evaluation sites (P1, P2, P3, TL1, TL2, TL3, TM1, TM2, and TM3). *, p≤0.05. The red line represents the Control group; the blue line represents the MSC group.


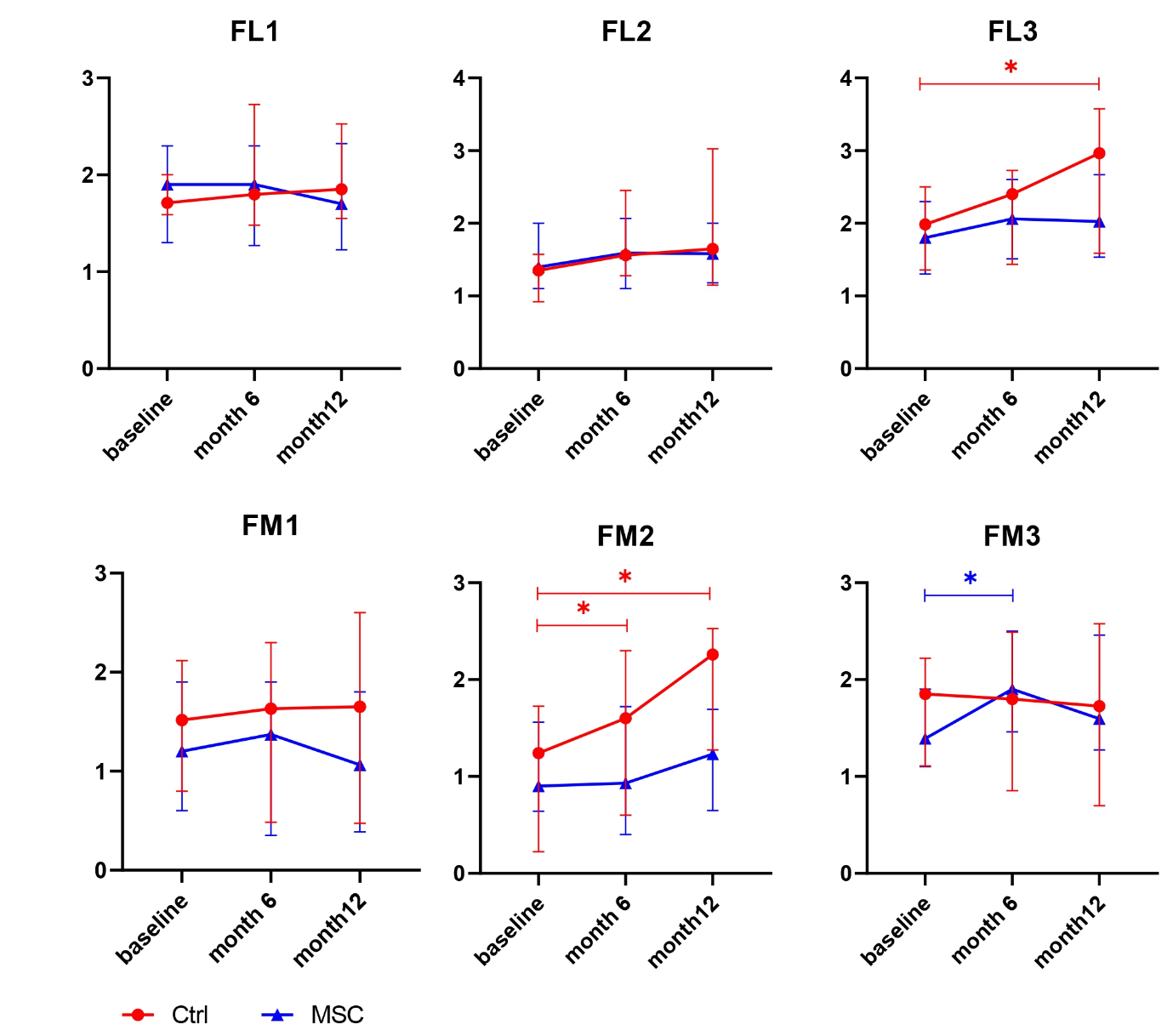


Figure S1 (continued).
