# Supplementary material for: Effectiveness and safety of multiple injections of human placenta-derived MSCs for knee osteoarthritis: a nonrandomized phase I trial
Source: BMC Musculoskelet Disord. 2025 Apr 26;26:418. doi: 10.1186/s12891-025-08664-2 (PMC12032682; doi:10.1186/s12891-025-08664-2)
Supplement: Supplementary file 2 — Supplementary Material 2 [file 12891_2025_8664_MOESM2_ESM.docx]

# **Supplementary materials**


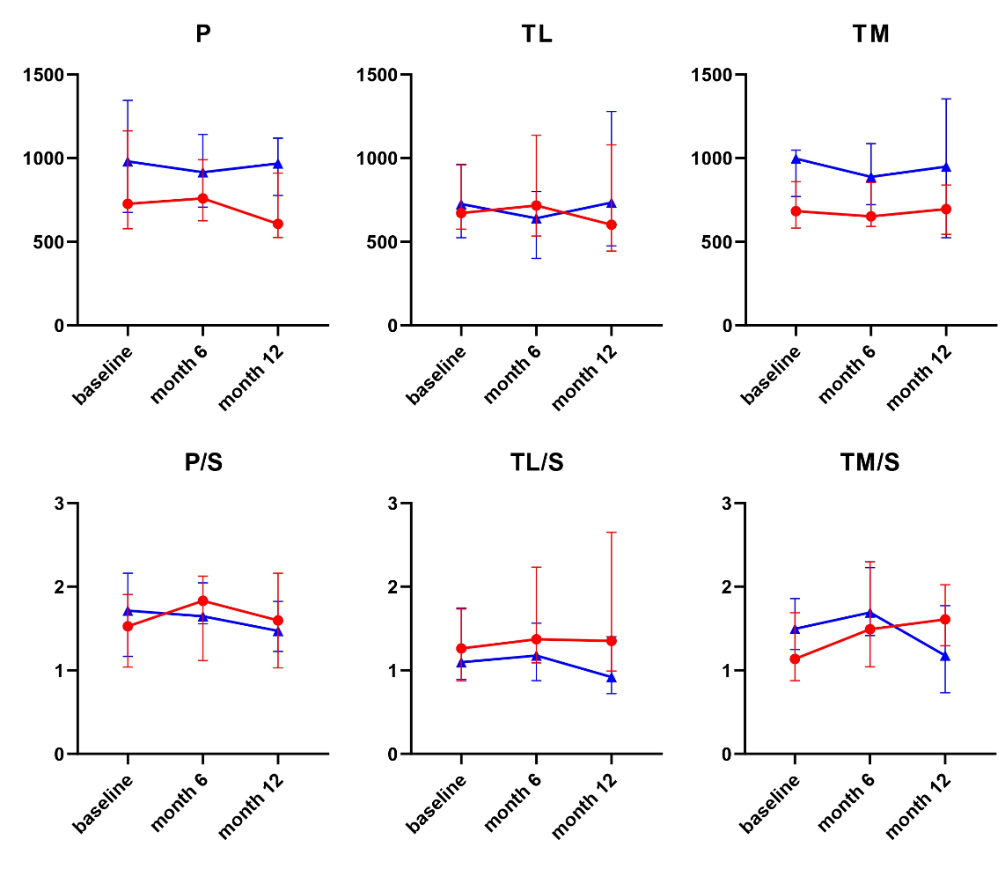


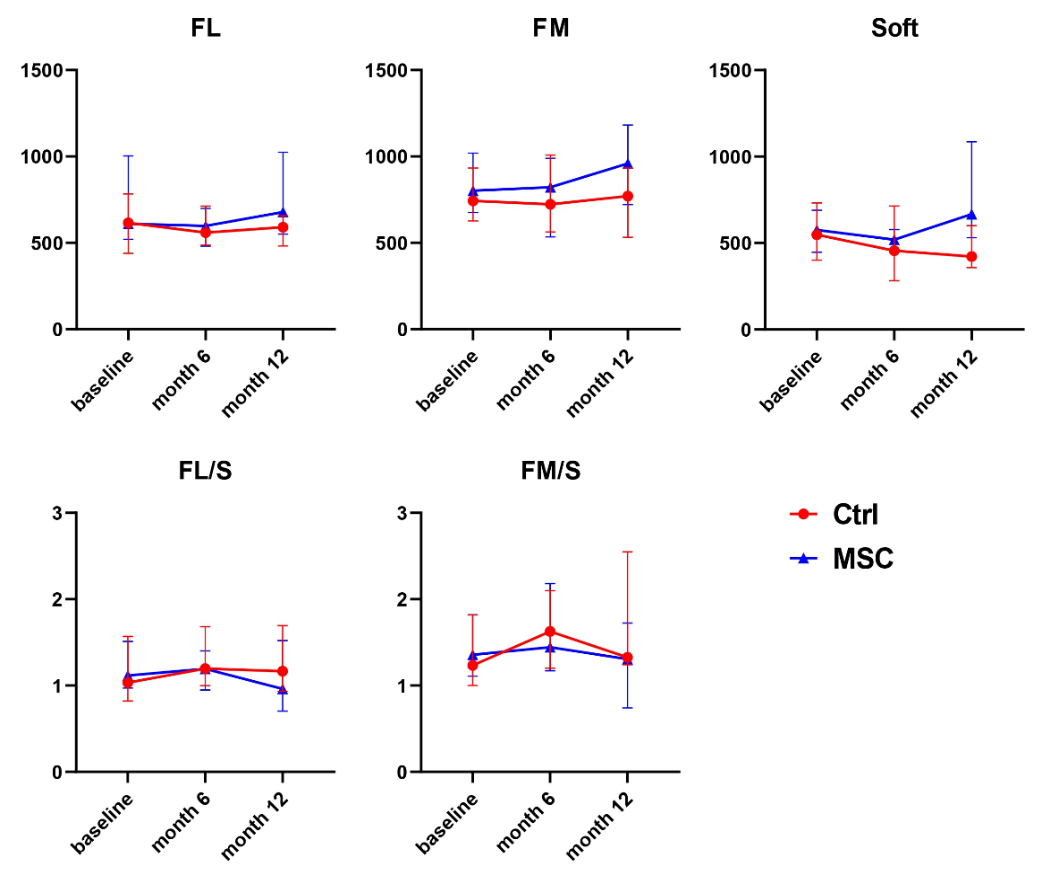


Figure S2. Cartilage optical density index by MRI analysis in patients with KOA in different evaluation sites (P, TL, TM, FL, FM). *, p≤0.05. The red line represents the Control group; the blue line represents the MSC group.
