# Supplementary material for: Effectiveness and safety of multiple injections of human placenta-derived MSCs for knee osteoarthritis: a nonrandomized phase I trial
Source: BMC Musculoskelet Disord. 2025 Apr 26;26:418. doi: 10.1186/s12891-025-08664-2 (PMC12032682; doi:10.1186/s12891-025-08664-2)
Supplement: Supplementary file 3 — Supplementary Material 3 [file 12891_2025_8664_MOESM3_ESM.docx]

# **Supplementary materials**


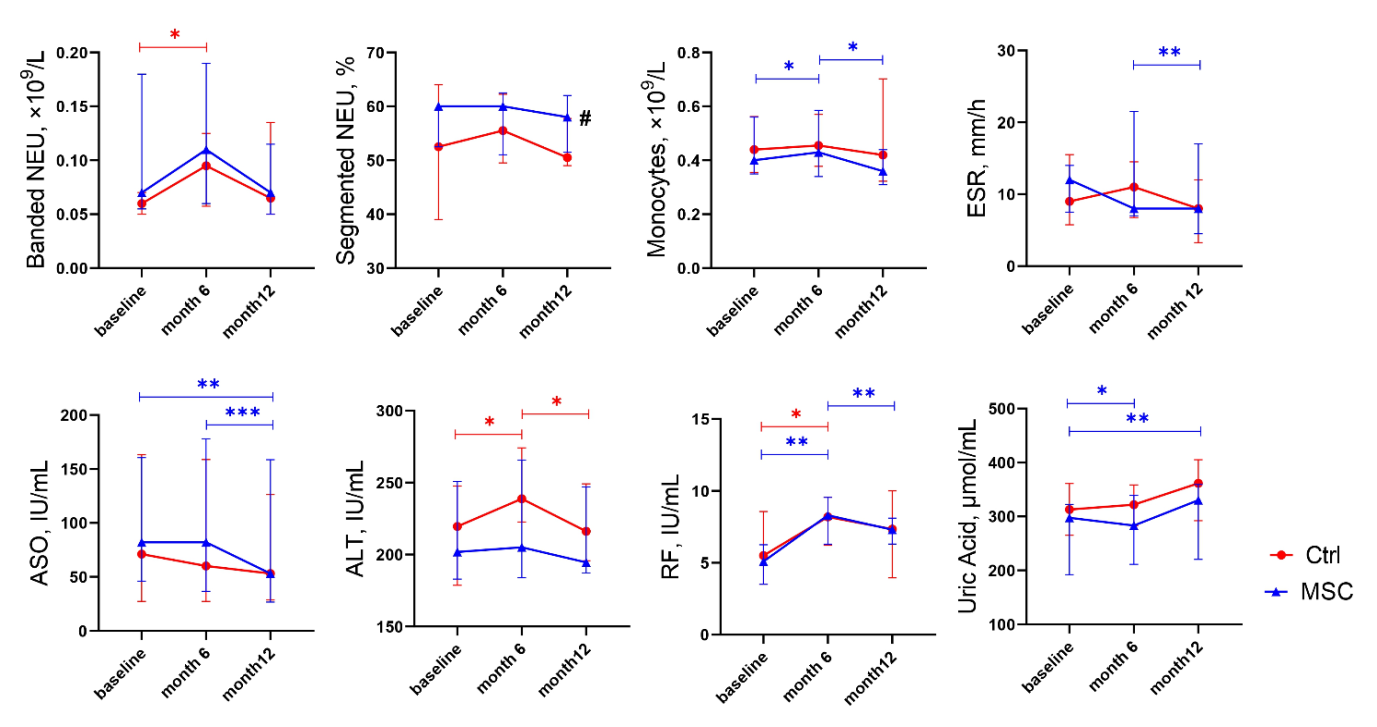


**Figure S3**. Hematological and biochemical parameters in patients with KOA. *, p≤0.05; **, p≤0.01; ***, p≤0.001; #, p≤0.05. ASO, antistreptolysin O; ALT, alanine transaminase; RF, rheumatoid factor; ESR, erythrocyte sedimentation rate. The red line represents the Control group; the blue line represents the MSC group.
